# Prognostic Value and Link to Atrial Fibrillation of Soluble Klotho and FGF23 in Hemodialysis Patients

**DOI:** 10.1371/journal.pone.0100688

**Published:** 2014-07-03

**Authors:** Albina Nowak, Björn Friedrich, Ferruh Artunc, Andreas L. Serra, Tobias Breidthardt, Raphael Twerenbold, Myriam Peter, Christian Mueller

**Affiliations:** 1 Division of Internal Medicine, University Hospital Zürich, Zürich, Switzerland; 2 Department of Internal Medicine, Division of Endocrinology, Diabetology, Vascular Disease, Nephrology and Clinical Chemistry, University of Tübingen, Tübingen, Germany; 3 Dialysis center Leonberg, Leonberg, Germany; 4 Division of Nephrology, University Hospital Zürich, Zürich, Switzerland; 5 Division of Nephrology, University Hospital Basel, Basel, Switzerland; 6 Division of Cardiology, University Hospital Basel, Basel, Switzerland; Institut National de la Santé et de la Recherche Médicale, France

## Abstract

Deranged calcium-phosphate metabolism contributes to the burden of morbidity and mortality in dialysis patients. This study aimed to assess the association of the phosphaturic hormone fibroblast growth factor 23 (FGF23) and soluble Klotho with all-cause mortality. We measured soluble Klotho and FGF23 levels at enrolment and two weeks later in 239 prevalent hemodialysis patients. The primary hypothesis was that low Klotho and high FGF23 are associated with increased mortality. The association between Klotho and atrial fibrillation (AF) at baseline was explored as secondary outcome. AF was defined as presence of paroxysmal, persistent or permanent AF. During a median follow-up of 924 days, 59 (25%) patients died from any cause. Lower Klotho levels were not associated with mortality in a multivariable adjusted analysis when examined either on a continuous scale (HR 1.25 per SD increase, 95% CI 0.84–1.86) or in tertiles, with tertile 1 as the reference category (HR for tertile two 0.65, 95% CI 0.26–1.64; HR for tertile three 2.18, 95% CI 0.91–2.23). Higher Klotho levels were associated with the absence of AF in a muItivariable logistic regression analysis (OR 0.66 per SD increase, 95% CI 0.41–1.00). Higher FGF23 levels were associated with mortality risk in a multivariable adjusted analysis when examined either on a continuous scale (HR 1.45 per SD increase, 95% CI 1.05–1.99) or in tertiles, with the tertile 1 as the reference category (HR for tertile two 1.63, 95% CI 0.64–4.14; HR for tertile three 3.91, 95% CI 1.28–12.20). FGF23 but not Klotho levels are associated with mortality in hemodialysis patients. Klotho may be protective against AF.

## Introduction

Cardiovascular mortality in hemodialysis patients is 55% higher than in patients with normal kidney function [1]. Vascular calcification has been linked to deranged calcium-phosphate metabolism. Fibroblast growth factor 23 (FGF23) regulates phosphate metabolism by inhibiting renal phosphate reabsorption [2], [3].

The transmembrane form of Klotho functions as co-receptor of FGF23 increasing the affinity of FGF23 to the FGF-receptor. Klotho is a 130-kDa single-pass transmembrane protein that is mainly expressed in the kidney [4]. The extracellular Klotho domain is cleaved into the circulation [5]. Klotho protects against arterial calcification [6] reduces arterial stiffness in chronic kidney disease (CKD) [7], increases endothelial survival [8]. Klotho expression decreases early in the course of CKD [9], [10] possibly causing FGF23 resistance [11].

High FGF23 levels were associated with an increased all-cause mortality in hemodialysis patients [12], increased risk for end stage renal disease (ESRD), cardiovascular events and mortality in CKD patients [13], [14]. An excess of FGF23 in CKD may be caused by low Klotho levels triggering FGFR’s peripheral resistance to FGF23 and thus linking FGF23-associated mortality to Klotho. To test this hypothesis, we assessed the association of soluble Klotho (sKlotho) with all-cause mortality as the primary outcome in a large cohort of hemodialysis patients.

Atrial fibrillation (AF) is one of the important cardiac comorbidities in hemodialysis patients. High FGF23 levels were associated with AF in a recent study [15]. Little is known of the association between low Klotho levels and AF. Experimental data demonstrated that sKlotho is important for the function of ion channels by regulating their cell-surface abundance through enzymatic activation [16]–[18]. This can influence the peacemaker activity of the channels in the sinoatrial node. Klotho’s expression in the sinoatrial node has been demonstrated in animal studies, Klotho-deficient animals develop sinoatrial dysfunction under stress conditions [19]. Thus, we tested a possible association between low Klotho level and AF.

## Subjects and Methods

This study was approved by the ethics committee of medical faculty Eberhard-Karls-university Tübingen (project 191/2009BO2). All study participants provided a written informed consent, both for taking of blood samples and for their clinical records to be used in the study. The research was done in accordance with the Helsinki declaration.

### Patients and control group

The study population consists of 239 prevalent maintenance hemodialysis patients from four dialysis centers in Southwest Germany participating in a prospective multicenter study. All the patients received bicarbonate hemodialysis. This cohort was established between September 2009 and September 2012 and investigate the associations between novel biochemical risk parameters and all-cause mortality [20], [21].

Patients were eligible if they had given written informed consent, initiation of hemodialysis was more than three months previously and there was no evidence of acute life-threatening illness, cardiac event, cardiac amyloidosis or cardiac procedure within the previous two months. Clinical data were collected at baseline from the patients’ medical records. The follow-up was complete in all patients.

### Endpoint evaluation

We defined all-cause mortality as the primary endpoint. The primary exposure variables were plasma Klotho and FGF23 levels, measured at study enrolment. Mortality was prospectively recorded and coded. The follow-up was conducted by the primary physicians of the cohort. Patients received status “survived” if they continued attending their regular thrice-weekly hemodialysis facility on 31.08.2012. No patient recovered kidney function. To ascertain the survival status of 18 patients who moved to another dialysis center, the new responsible primary physician was contacted. The follow-up of 14 transplanted patients was censored at the transplantation date.

The association between sKlotho and the prevalence of AF at baseline was explored as a secondary outcome in a cross-sectional manner. The diagnosis of AF was extracted from patient records. In this cohort, 12-lead ECGs were routinely performed every 6 months and additionally if patients complained of palpitations or chestpain. If no AF was seen on the 12-lead ECG, a 24-hour ECG was recorded promptly. AF was defined as the presence of paroxysmal, persistent or permanent AF.

### Control group

The Klotho control group consisted of 80 healthy blood donors who permitted use of their samples for research purposes (age 45±13 years). Blinded to Klotho, we generated age-adjusted groups by excluding hemodialysis patients older than 70 and controls younger than 50 years. The age-adjusted groups included 109 hemodialysis patients aged 56±11 and 32 controls aged 56±4 years.

The FGF23 control group consisted of 55 healthy blood donors who permitted use of their samples for research purposes. Blinded to FGF23, we generated age-adjusted groups by including all dialysis patients in the cohort and controls older than 60 years. The age-adjusted groups included 239 hemodialysis patients aged 68±14 and 19 controls aged 69±8 years.

### Laboratory assays

Blood was drawn prior to the dialysis session at enrolment and two weeks later, collected in EDTA, cooled to 4°C, centrifuged within 4 hours, the plasma samples were stored at −80°C for further batched analysis. Klotho and FGF23 levels were measured in a blinded fashion.

Human soluble α-Klotho levels were measured by Enzyme Linked Immunosorbent Assay (ELISA) (Immuno-Biologic Laboratories Co., Ltd. Japan). This novel method detects sKlotho using a monoclonal antibody with high affinity to the human α-Klotho protein. According to the manufacturer’s manual, the intra assay variation coefficients (VC) are 2.7% and 3.1% at 757 pg/ml and 2969 pg/ml, the interassay VC are 6.5% and 2.9% at 706 pg/mL and 2903 pg/mL, respectively; the analytic sensitivity is ≥6.15 pg/mL. Plasma FGF23 concentrations were measured using the two-site second generation Human FGF23 (C-Term) ELISA Kit (Immutopics, San Clemente, CA) with mean intra-assay VC <3%, as specified by the manufacturer. We additionally measured FGF23 levels in 36 patients using Millipore human FGF23 (intact) ELISA Kit (Billerica, MA, USA) and in 32 patients using Kainos (Tokyo, Japan) murine FGF23 (intact) ELISA Kit. To verify the capability of the chosen FGF23 and Klotho assays, we performed dilution experiments in 11 random samples. We tested sample: diluent ratios 1∶2 and 1∶4. The correlation between measured and expected Klotho and FGF23 levels in the diluted samples was very high: R = 0.97, P>0.001 for Klotho and R = 0.99, P<0.001 for FGF23 (Figure S1 in File S1).

Due to low sample volume in some patients, we first measured FGF23 as it required a lower sample volume, then measuring Klotho if enough volume remained.

Laboratory values of PTH, hemoglobin, albumin, C-reactive protein (CRP), calcium, phosphate and alkaline phosphatase were extracted from the patient medical records and averaged from the available values of the previous year (4–12 values). 25(OH)vitamin D levels were measured between 3 months before and 3 months after study enrolment and were available in 223 patients Body Mass Index (BMI) was calculated as postdialytic dry weight at study enrolment in kilograms divided by the square of the height in meters.

### Clinical data

All clinical evaluation of the patients was performed at the time the prospective cohort was established. Data on residual diuresis (measured by 24 h urine collection and quantified in milliliters per day), single pool Kt/V (mean of the last 4 values), interdialytic weight gain, predialytic blood pressure (means from the last 12 values), dialysis access and membrane, time on dialysis, blood pump, arterio-venous fistula flow (measured with a Transonic system, Ithaca, NY, USA), presence of pulmonary hypertension (using echocardiography or right heart catheter as indicated) were extracted from the medical records. Transthoracic echocardiography (TTE) was performed as clinically indicated. The results of TTE performed within one year before and one year after the study enrolment were extracted from the medical records and analysed for the presence of left ventricular hypertrophy (LVH) and left atrium dilation (LAD). These results were available in166 patients for LVH and in 177 for LAD.

### Statistical analysis

We used descriptive statistics to compare baseline characteristics and laboratory parameters according to the baseline Klotho tertiles. Categorical variables were expressed as proportions, continuous variables as means with standard deviations and medians with interquartile ranges [IQR]. Normal distribution was assessed by Kolmogorov-Smirnov-Test. Comparisons of groups were made using one-way analysis of variance (ANOVA) for continuous and the chi-squared test for categorical variables.

Kaplan-Meier analysis was performed and log-rank test of survival distributions equality for the Klotho and FGF23 tertiles was calculated.

Cox regression analysis was used to examine the mortality risk associated with baseline Klotho and FGF23 levels. Similarly to previous studies examining FGF23 and mortality risk in CKD [13], [14] and hemodialysis [12], multivariable models were applied to adjust for potential confounders, using prior knowledge of variables that have been associated with mortality risk in patients undergoing hemodialysis in previous studies. We hierarchically adjusted for demographics (age, gender, dialysis center) in model 1, dialysis-specific risk factors and comorbid conditions (dialysis vintage, blood pressure, BMI, vascular access at study enrolment, diabetes mellitus, coronary and valvular heart disease, AF, ICD carriage, peripheral and cerebrovascular disease, COPD, malignancy, cause of renal failure, medication and pooled Kt/V) in model 2, and mineral metabolism levels (PTH, 25(OH)vitamin D, phosphate and calcium), albumin, hemoglobin, CRP, cholesterol in model 3.

We used logistic regression analysis to explore the association between baseline Klotho levels and AF. Multivariable models were applied to adjust for potential confounders, using variables that have been associated with AF in previous studies. We hierarchically adjusted for demographics (age, gender, dialysis center) in model 1, cardiovascular comorbidities (diabetes mellitus, coronary artery and valvular heart disease, peripheral artery and cerebrovascular disease), anuria in model 2, and mineral metabolism levels (FGF23, PTH, phosphate, calcium), calcium dialysate, potassium serum and dialysate, albumin, hemoglobin, CRP, cholesterol, thyroid stimulating hormone in model 3. Regression analysis for Klotho tertiles at baseline with the absence of AF as dependent variable was performed using the same models.

The statistical analyses were performed using the SPSS/PC (version 19.0; SPSS Inc., Chicago, IL, USA) software package. Numbers at risk were calculated using SAS 9.3 (SAS Institute, Cary, NC, USA). All statistical tests were two-sided, and P values <0.05 were considered significant.

## Results

### Baseline characteristics and outcome

From a total of 250 eligible patients, 239 (96%) were enrolled in our study. The exclusion reasons were: six declined to participate, two died within two weeks after enrolment, two suffered from cardiac amyloidosis and one had initiated hemodialysis less than three months before. The follow-up was complete in all patients. Fifty-nine patients (25%) died within the median follow-up period of 924 [735–996] days.

Klotho at enrolment was measured in 55 (93%) non-survivors and 172 (96%) survivors, FGF23 in 57 (97%) non-survivors and 177 (98%) survivors. Both, Klotho and FGF23 levels at baseline were measured in 226 (95%) patients. The baseline characteristics and laboratory results for the full cohort and according to the Klotho level tertiles are given in Table 1.

**Table 1:** Baseline characteristics and laboratory parameters according to Klotho tertiles.

	Full Cohort	Klotho Tertile 1	Klotho Tertile 2	Klotho Tertile 3	P value
	339 [260–427]N = 239	(<286 pg/ml) N = 75	(286–392 pg/ml)N = 75	(>392 pg/ml)N = 77	
Age (yr)	68±14	67±15	69±12	66±15	0.42
Male gender n (%)	153 (64)	55 (73)	41 (55)	49 (64)	0.06
Body-mass index*	26 [23]–[30]	27 [24]–[30]	26 [23]–[29]	26 [22]–[30]	0.41
Blood pressure (mmHg)					
Systolic	134 [122–144]	137 [128–147]	134 [124–144]	127 [117–140]	0.005
Diastolic	69 [63–74]	69 [63–76]	68 [64–74]	69 [61–73]	0.55
Cause of renal failure n (%)					
Diabetes mellitus	63 (26)	24 (36)	19 (25)	18 (23)	0.48
Hypertension	19 (8)	5 (7)	8 (11)	3 (4)	0.26
Glomerulonephritis	71 (30)	24 (32)	23 (31)	22 (29)	0.90
PKD	11 (5)	2 (3)	5 (7)	4 (5)	0.51
Others/unknown	75 (31)	20 (27)	20 (27)	30 (39)	0.17
Cardiac comorbidities n (%)					
Coronary artery disease	73 (31)	24 (32)	24 (32)	19 (25)	0.52
PCI/CABG	45 (19)	16 (21)	19 (25)	17 (22)	0.78
Valvular heart disease	61 (26)	13 (17)	20 (27)	22 (29)	0.27
Atrial fibrillation	54 (23)	19 (25)	20 (27)	10 (13)	0.08
ICD implantation	4 (2)	1 (1)	0 (0)	2 (3)	0.37
LVH†	101 (61)	36 (62)	34 (62)	28 (52)	0.18
Pulmonary hypertension	16 (7)	3 (4)	6 (8)	5 (6)	0.59
Other comorbidities n (%)					
Diabetes mellitus	90 (38)	29 (39)	24 (32)	32 (43)	0.46
PVD	80 (33)	28 (37)	23 (31)	23 (31)	0.56
Stroke	38 (16)	8 (11)	18 (24)	9 (12)	0.04
Vasculitis	8 (3)	3 (4)	2 (3)	2 (3)	0.85
Malignoma	34 (14)	14 (19)	7 (9)	11 (14)	0.26
COPD	19 (8)	3 (4)	8 (11)	7 (9)	0.29
Dialysis vintage (months)	59±53	49±40	67±56	62±60	0.10
Duration of dialysis session (hours)	4.2±0.4	4.3±0.5	4.1±0.3	4.2±0.42	0.24
Dialysis access at baseline n (%)					
Arteriovenous fistula	169 (71)	49 (65)	54 (72)	56 (73)	0.51
PTFE graft	31 (13)	11 (15)	7 (9)	11 (14)	0.57
Tunnelled catheter	38 (16)	15 (20)	13 (17)	10 (13)	0.50
Dialysis membrane n (%)					
High-flux	228 (95)	73 (97)	70 (93)	73 (95)	0.51
Low-flux	11 (5)	2 (3)	5 (7)	4 (5)	
Dialysis modality					
Hemodiafiltration	54 (23)	18	17	15	0.79
Hemodialysis only	185 (77)	57	58	62	
Anuric patients n (%)	93 (39)	28 (37)	30 (40)	32 (42)	0.86
Residual diuresis (ml/24 hours)	250 [0–1005]	300 [0–1100]	200 [0–1025]	200 [0–900]	0.75
Interdialytic weight gain (kg)	1.9 [1.3–2.5]	1.9 [1.4–2.7]	1.82 [1.22–2.43]	1.86 [1.17–2.48]	0.52
Shunt flow (ml/min)	1177±638	1099±512	1287±670	1160±694	0.28
Singe pool Kt/V	1.6 [1.4–1.7]	1.52 [1.33–1.65]	1.60 [1.40–1.77]	1.53 [1.39–1.71]	0.24
Ca, dialysate	1.4±0.2	1.4±0.2	1.4±0.2	1.4±0.2	0.98
K, dialysate	2.4±0.6	2.3±0.6	2.3±0.6	2.5±0.6	0.40
FGF23 (RU/ml)	883±1940	1117±2866	609±1025	761±1370	0.25
PTH (pg/ml)	249±177	282±184	240±188	234±152	0.20
25(OH)vitamin D (ng/ml)	28 [21]–[35]	28 [20]–[35]	27 [21]–[34]	29 [20]–[36]	0.52
Phosphate (mmol/l)	1.6 [1.4–1.9]	1.6 [1.3–1.9]	1.6 [1.3–1.8]	1.6 [1.4–1.9]	0.70
Ca, serum (mmol/l)	2.3 [2.2–2.4]	2.3 [2.2–2.4]	2.3 [2.2–2.4]	2.3 [2.2–2.4]	0.40
AP (U/l)	91±42	85±41	91±43	97±45	0.14
K, serum (mmol/l)	5.0±0.6	5.1±0.6	5.1±0.6	5.0±0.6	0.37
Albumin (g/l)	37±4	37±5	37±4	37±4	0.82
Hemoglobin (g/dL)	11.5 [11.1–12.0]	11.5 [11.2–11.8]	11.6 [11.2–12.0]	11.5 [11.2–12.0]	0.9
C-reactive protein (mg/l)	12±12	13±14	12±11	12±11	0.80
Cholsterol (mg/dl)	167 [141–196]	171 [147–200]	172 [143–199]	156 [141–193]	0.52
Medication use n (%)					
Phosphate binders	205 (86)	64 (85)	64 (85)	67 (87)	0.88
Vitamin d replacement	233 (97)	72 (96)	74 (99)	75 (97)	0.76
ACE-I or ARB	126 (53)	48 (64)	39 (52)	33 (43)	0.04
β-Blockers	153 (64)	56 (75)	45 (60)	42 (55)	0.03
Statin	101 (42)	41 (55)	28 (37)	26 (34)	0.02

Plus-minus values are means ± SD. Numbers with ranges in square brackets are medians and interquartile ranges. P values are for the comparisons between the three Klotho tertiles. To convert the values for calcium to milligrams per deciliter, multiply by 4.000. To convert the values for phosphate to milligrams per deciliter, multiply by 3.0969.

*The body-mass index is the weight in kilograms divided by the square of the height in meters.

† Based on 166 available clinically indicated transthoracic echocardiography results.

**Abbreviations:** ACE-I, angiotensin-converting enzyme inhibitors; AF, atrial fibrillation; AP, alkaline phosphatase; ARB, angiotensin receptor blocker; COPD, chronic obstructive pulmonary disease; FGF23, fibroblast growth factor 23; ICD, implantable cardioverter defibrillator; IQR, interquartile range; PTFE, Polytetrafluorethylen; PTH, parathyroid hormone; PKD, polycystic kidney disease; PVD Peripheral vascular disease; T, Tertile; U, unit.

### All-cause mortality

Median Klotho levels were similar in non-survivors and survivors (351 [264–454] vs 338 [255–420] pg/ml, P = 0.85). Median FGF23 levels tended to be higher in non-survivors than in survivors (394 [111–1147] vs 201 [83–707] RU/ml, P = 0.07).

In Cox regression analysis, higher Klotho levels were not associated with mortality in crude model and after multiple adjustments when examined either on a continuous scale or in tertiles, with tertile 1 as the reference category. In contrast, higher FGF23 levels were associated with mortality after the same adjustments (Table 2). Similar results were obtained analysing the second blood sample (Table S1 in File S1).

**Table 2:** Hazard Ratios (and 95% CIs) for Death per Standard Deviation of FGF23 and Klotho levels and according to the level tertiles.

Parameter	Crude			Model 1*			Model 2†			Model 3‡		
	HR	95%CI	P value	HR	95%CI	P value	HR	95%CI	P value	HR	95%CI	P value
Klotho (N = 227)	1.03	0.77–1.34	0.85	1.03	0.78–1.36	0.83	1.10	0.76–1.60	0.62	1.30	0.86–1.96	0.22
Klotho§												
Tertile 1 (N = 75)	R			R			R			R		
Tertile 2 (N = 75)	0.79	0.40–1.57	0.50	0.67	0.33–1.33	0.25	0.55	0.23–1.31	0.18	0.68	0.27–1.74	0.42
Tertile 3 (N = 77)	1.22	0.66–2.28	0.53	1.19	0.63–2.22	0.59	1.70	0.74–3.88	0.21	2.42	1.00–5.87	0.05
FGF23 (N = 234)	1.36	1.09–1.70	0.006	1.39	1.12–1.74	0.003	1.40	1.04–1.89	0.03	1.59	1.13–2.22	0.007
FGF23||												
Tertile 1 (N = 77)	R			R			R			R		
Tertile 2 (N = 78)	0.93	0.46–1.91	0.85	0.98	0.48–2.01	0.95	1.61	0.67–3.89	0.29	1.75	0.69–4.44	0.24
Tertile 3 (N = 79)	1.82	0.97–3.43	0.06	2.17	1.15–4.09	0.02	2.65	1.09–6.44	0.03	3.73	1.15–12.11	0.03

*Model 1 = demographics: adjusted for age, gender (male) and by dialysis center clustering.

† Model 2 = dialysis specific risk factors and comorbid conditions: adjusted for covariates in Model 1 plus dialysis vintage, systolic and diastolic blood pressure, body-mass index, vascular access on study enrolment (fistula, graft, catheter), coexisting conditions listed in Table 1 (coronary artery disease, valvular heart disease, atrial fibrillation, pulmonary hypertension, implantable cardioverter defibrillator carrier; diabetes mellitus, peripheral vascular disease, stroke, vasculitis, malignoma, chronic obstructive pulmonary disease), cause of renal failure (diabetic nephropathy, hypertensive nephropathy, glomerulonephritis, polycystic kidney disease, others/unknown), medication use listed in Table 1 (phosphate binders, vitamin D replacement, angiotensin converting enzyme inhibitors or angiotensin receptor blockers, beta blockers, aspirin, anticoagulant or clopidogrel, statin), pooled Kt/V.

‡ Model 3 = fully adjusted model: adjusted for covariates in Model 2 plus parathyroid hormone, 25(OH)vitamin D, phosphate, calcium, albumin, hemoglobin, C-reactive protein, cholesterol.

§ Patients were categorized according to Klotho level tertiles at enrolment (1^st^ tertile <286 pg/ml, 2^nd^ tertile 286–392 pg/ml, 3^rd^ tertile >392 pg/ml).

|| Patients were categorized according to FGF23 level tertiles at enrolment (1^st^ tertile <118 RU/ml, 2^nd^ tertile 118–468 RU/ml, 3^rd^ tertile >468 RU/ml).

Abbreviations: FGF23, fibroblast growth factor 23; HR, hazard ratio; R, reference.

Following adjustment for demographics and markers of bone metabolism, haemoglobin, albumin and cholesterol, Klotho remained not associated with mortality (HR 1.21 per SD increase, 95% CI 0.87–1.68, P = 0.25), in contrast to FGF23 (HR 1.56 per SD increase, 95% CI 1.21–2.01, P = 0.001).

**Figure 1:**
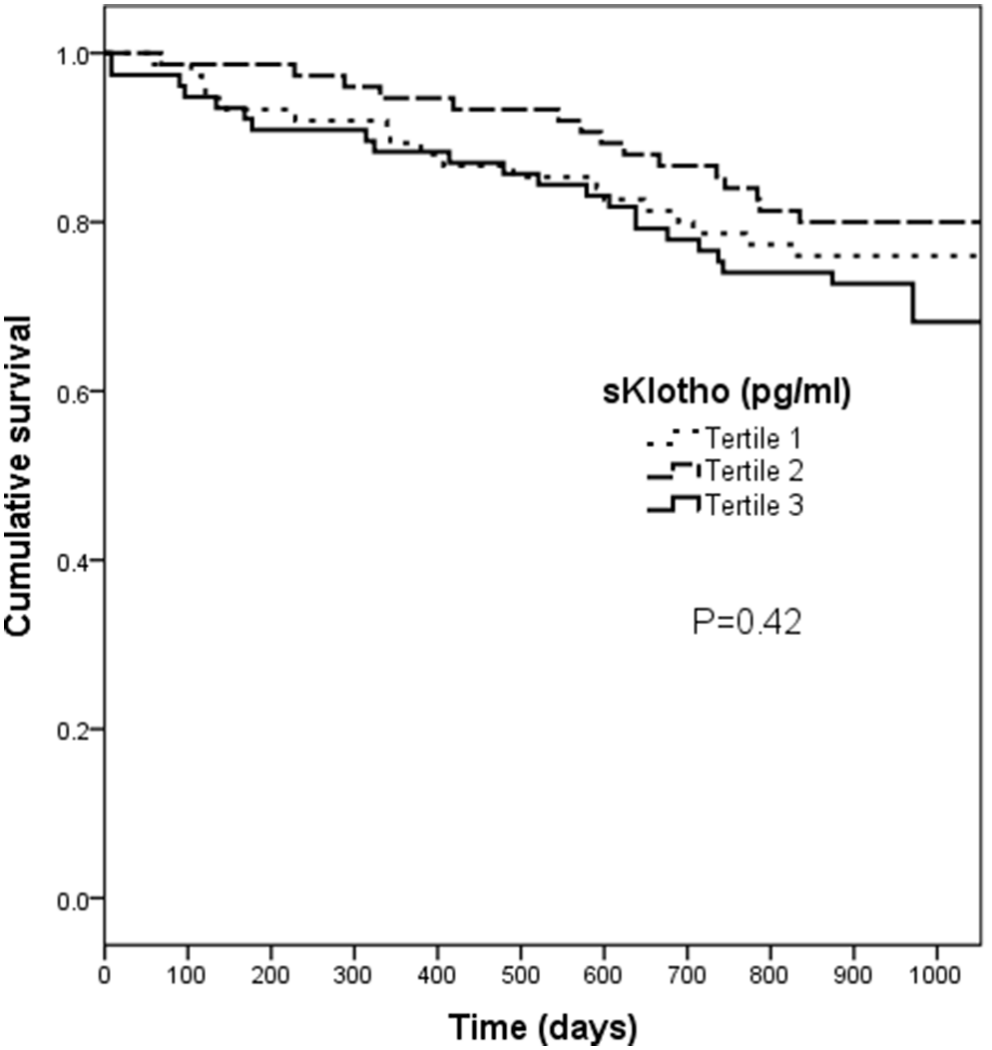
Cumulative survival by tertiles of secreted Klotho. Patients were stratified by their baseline Klotho levels according to the tertiles. Kaplan-Meier analysis with long-rank test did not reveal a significant difference between groups (P = 0.42).

**Figure 2:**
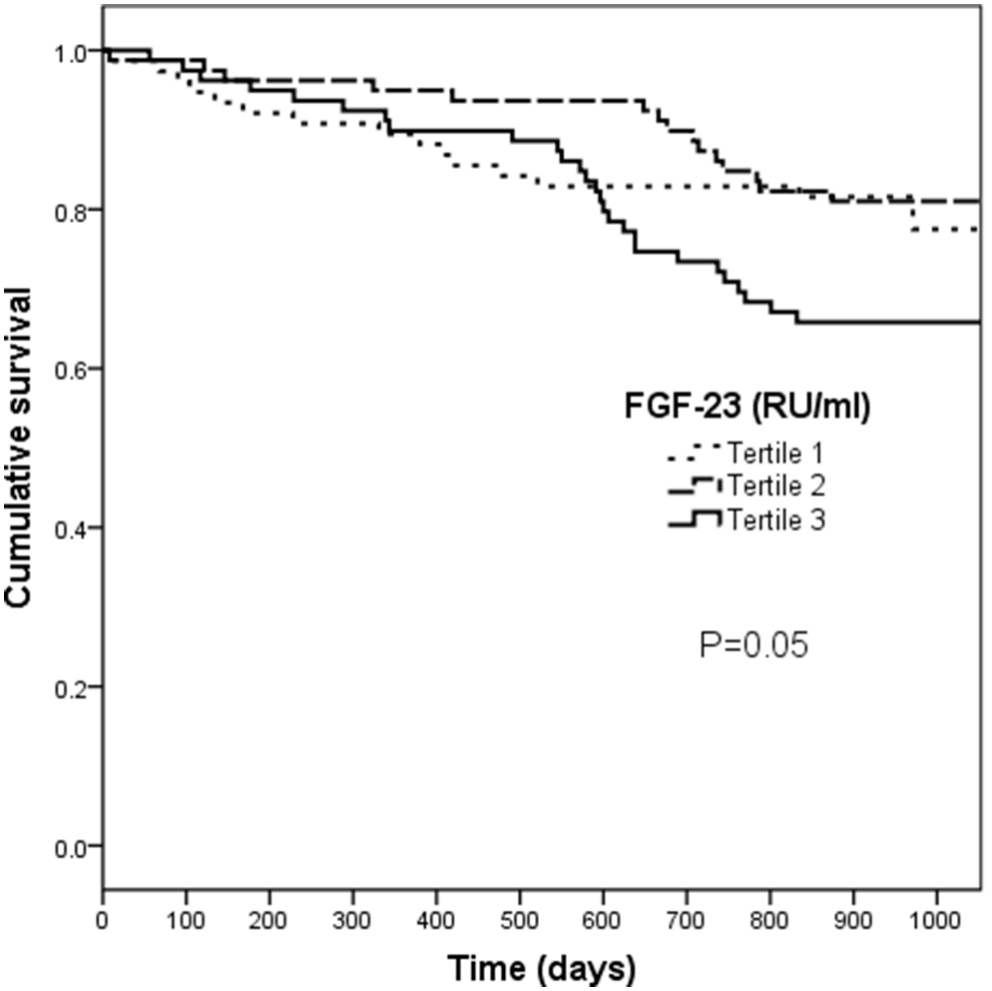
Cumulative survival by tertiles of Fibroblast growth factor 23 (FGF23). Patients were stratified by their FGF23 levels according to the tertiles. Kaplan-Meier analysis with long-rank test approached a significant difference between groups (P = 0.05).

Cumulative survival among the three groups, stratified according to the baseline Klotho tertiles, was similar in a Kaplan-Meier analysis (P = 0.42) (Figure 1). For FGF23, the poorest cumulative survival was observed among patients with levels within the top tertile, with a log-rank test approaching significance (P = 0.05) (Figure 2).

There was no correlation between FGF23 and Klotho levels (R = −0.03, P = 0.64).

The ratio between FGF23 (RU/ml) per SD increase and Klotho (pg/ml) per SD increase did not substantially improve the association with mortality (HR 1.59 95% CI 1.16–2.19, P = 0.004) compared to FGF23 per SD increase alone in a crude Cox regression model.

### Atrial fibrillation

In 54 (23%) of 239 patients, AF had been diagnosed. The baseline characteristics and laboratory results according to presence or absence of AF are presented in Table S2 in File S1.

**Figure 3:**
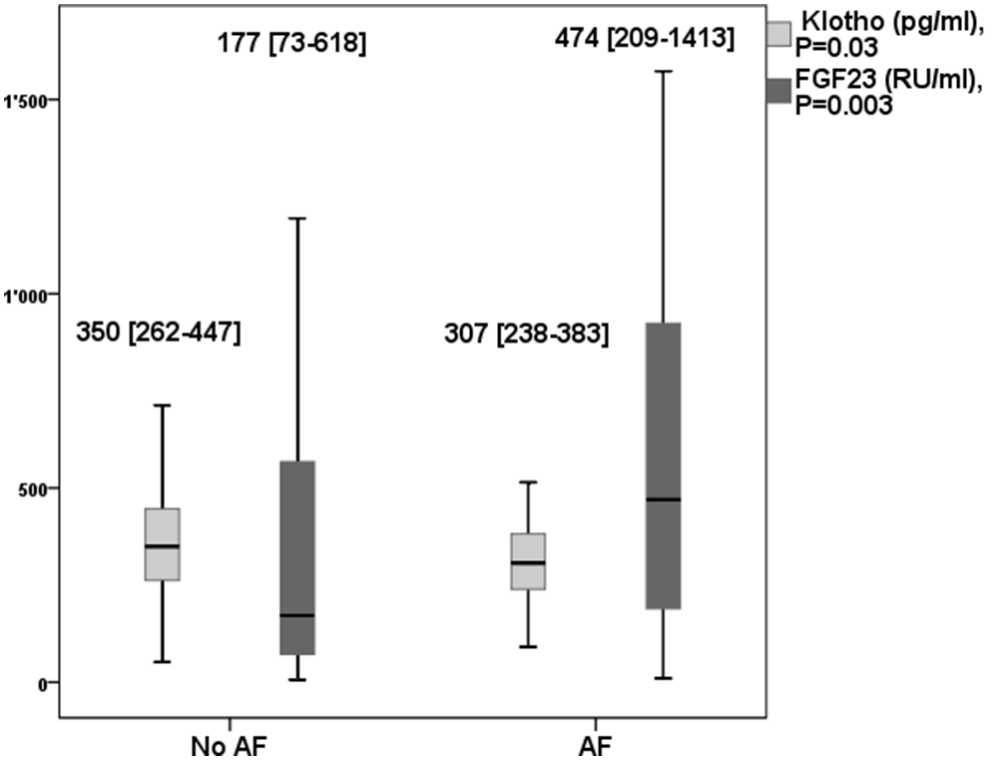
Klotho and FGF23 levels in patients with and without atrial fibrillation.

Both low Klotho and high FGF23 levels were associated with AF (Figure 3). In logistic regression analysis, low Klotho levels remained associated with AF in crude model, in model 1, model 2, this association was slightly attenuated in model 3 (Table 3).

**Table 3:** Association of Klotho levels with the presence of atrial fibrillation.

Parameter	Crude			Model 1*			Model 2†			Model 3‡		
	OR	95%CI	P value	OR	95%CI	P value	OR	95%CI	P value	OR	95%CI	P value
Klotho§ (pg/mL)	0.60	0.52–0.68	<0.001	0.45	0.31–0.65	<0.001	0.43	0.29–0.64	<0.001	0.60	0.38–0.95	0.03
Age (years)				1.01	1.01–1.04	0.08	1.00	0.98–1.01	0.59	1.07	1.03–1.11	0.002
Gender (male)				0.83	0.44–1.59	0.58	0.76	0.36–1.61	0.48	1.26	0.48–3.27	0.64
DM							0.85	0.36–2.01	0.71	0.08	0.41–2.84	0.88
CAD							2.96	1.37–6.37	0.006	2.37	1.00–5.63	0.05
VHD							4.05	1.82–9.02	0.001	3.85	1.51–9.62	0.004
PAD							1.80	0.78–4.14	0.17	1.43	0.57–3.63	0.45
Stroke							1.14	0.45–2.89	0.78	1.11	0.38–3.26	0.84
Anuria							2.07	1.02–4.22	0.04	2.38	1.37–8.32	0.008
PTH (pg/ml)										1.00	1.00–1.00	0.79
FGF23§ (RU/ml)										1.65	0.86–3.17	0.13
Phosphate (mmol/l)										0.40	0.12–1.48	0.17
Ca, serum (mmol/l)										0.28	0.02–4.07	0.35
Ca, dialysate (mmol/l)										0.90	0.01–1.14	0.06
K, serum (mmol/l)										1.22	0.62–2.40	0.56
K, dialysate (mmol/l)										1.44	0.70–2.93	0.32
Albumin (g/l)										1.03	0.91–1.17	0.67
Hb (g/dl)										0.88	0.51–1.51	0.67
CRP (mg/l)										1.02	0.98–1.05	0.32
Cholesterol (mg/dl)										1.00	0.99–1.01	0.61
TSH (mU/l)										1.15	0.86–1.53	0.35

*Model 1: adjusted for age and gender (male).

† Model 2: adjusted for covariates in Model 1 plus cardiovascular comorbidities and anuria.

‡ Model 3: adjusted for covariates in Model 2 plus laboratory results for mineral metabolism, calcium dialysate, potassium serum and dialysate, inflammation, cholesterol, hemoglobin and TSH.

§ per standard deviation.

Abbreviations: Ca, Calcium; CAD, coronary artery disease; CI, confidence interval; CRP, C-reactive protein; DM, diabetes mellitus; FGF23, fibroblast growth factor 23; Hb, hemoglobin; K, potassium; PAD, peripheral artery disease; TSH, thyroid stimulating hormone.

OR, odds ratio; VHD, valvular heart disease.

This association was similar if we adjusted only for demographics and markers of bone metabolism, haemoglobin, albumin and cholesterol (HR 0.66 per SD increase, 95% CI 0.43–1.00, P = 0.05).

Patients with Klotho levels within the third tertile were more frequently free of AF (Table 4). This effect remained significant after multiple adjustments. A similar association was observed analysing the results of the second blood sample (Tables S3 and S4 in File S1).

**Table 4:** Regression analysis for Klotho tertiles with the absence of atrial fibrillation.

	Klotho Tertile 1,N = 75 (<286 pg/ml)	Klotho Tertile 2,N = 75 (286–392 pg/ml)		Klotho Tertile 3,N = 77 (>392 pg/ml)	
		OR (95%CI)	P value	OR (95%CI)	P value
Crude	Reference	2.75 (1.65–4.59)	<0.001	6.70 (3.45–13.02)	<0.001
Model 1*	Reference	1.09 (0.52–2.27)	0.83	2.90 (1.27–6.64)	0.01
Model 2†	Reference	1.23 (0.54–2.78)	0.63	3.71 (1.49–9.25)	0.005
Model 3‡	Reference	1.00 (0.40–2.59)	1.00	3.02 (1.03.89–8.82)	0.04

*Model 1: adjusted for age and gender.

† Model 2: adjusted for covariates in Model 1 plus diabetes mellitus, coronary artery disease, valvular heart disease, peripheral vascular disease, stroke and anuria.

‡ Model 3: adjusted for covariates in Model 2 plus parathyroid hormone, fibroblast growth factor 23, calcium, phosphate, albumin, calcium dialysate, potassium serum and dialysate, hemoglobin C-reactive protein, cholesterol and thyroid stimulating hormone.

Abbreviations: CI, confidence interval; OR, odds ratio.

The presence of AF tended to be associated with mortality in a crude Cox regression model (HR 1.67; 95%CI 0.97–2.89; P = 0.07).

### Klotho and FGF23 levels in control group versus hemodialysis patients

**Figure 4:**
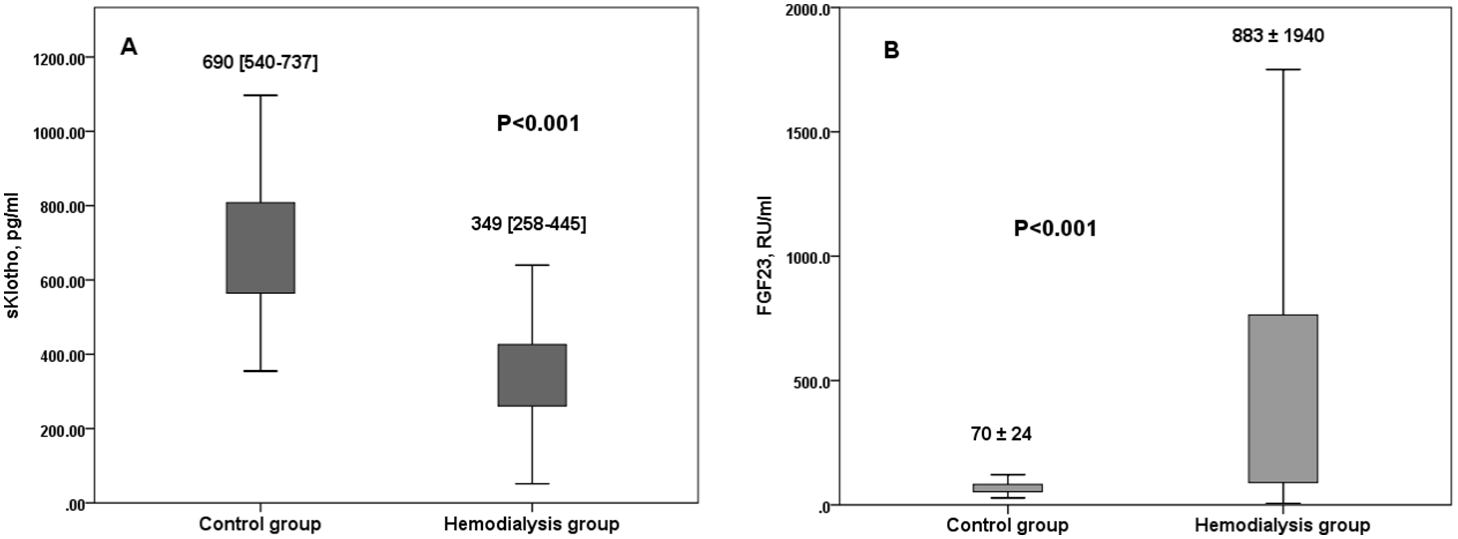
Klotho (A) and FGF23 (B) levels in controls versus hemodialysis patients.

The median Klotho level in controls was higher than in hemodialysis patients before (Figure 4A) and after age adjustment (647 [381–974], versus 344 [116–752] pg/ml, P<0.001). Klotho levels correlated with age in controls (R^2^ = −0.293, P = 0.008) but not in haemodialysis patients (P = 0.78).

The mean FGF23 level in controls was lower than in haemodialysis patients before (Figure 4B) and after age adjustment (93±25, versus 883±1940 RU/ml, P = 0.001). FGF23 levels did not correlate with age in controls (P = 0.11) and in hemodialysis patients (P = 0.17).

### FGF23 levels measured with different kits

The mean FGF23 level measured using the Millipore assay was 1028±2119 pg/ml, using Immutopics assay in the same samples 1001±1627 RU/ml. The mean FGF23 level measured using Kainos assay was 690±1001 pg/ml, using the Immutopics assay in the same samples 723±1279 RU/ml.

FGF23 levels measured using the Immunotopic assay correlated with the levels measured using the Millipore (R = 0.73, P<0.001) and Kainos assay (R = 0.66, P<0.001). FGF23 levels measured using Millipore assay correlated with the levels measured using Kainos assay (R = 0.97, P<0.001).

## Discussion

In this cohort of ESRD patients with long-term follow-up, we report three major findings. First, FGF23 but not Klotho levels were predictive for all-cause mortality. This association remained significant after multivariate adjustments and was confirmed analysing the results of the second blood sample drawn two weeks after study enrolment. Second, high Klotho levels may exert a protective effect against AF. Interestingly, Klotho was more strongly associated with AF than the presence of traditional cardiovascular risk factors such as complete loss of kidney function, age, gender, arteriosclerosis and valvular heart disease. Even the fully adjusted model, additionally including levels of other mineral metabolites known as risk factors for structural heart disease, approached statistical significance. Third, Klotho levels in healthy controls were higher than in hemodialysis patients.

Assays for Klotho have been lacking until now, and data on the regulatory mechanism, function and expression of Klotho has been scant. Only very recently, a sandwich enzyme-linked immunosorbent assay (ELISA) has been developed for the soluble form of Klotho [22]. Using this, we determined Klotho and FGF23 blood levels in a large multicentre cohort of hemodialysis patients and tested the hypothesis that low blood Klotho levels could be associated with all-cause mortality. To our knowledge, this is the first study exploring an association of Klotho with all-cause mortality in hemodialysis patients.

Although Klotho has been shown to exert direct vasculoprotective effects, our data extend recent observations by Seiler et al in patients with stage 2–4 chronic kidney disease which showed Klotho levels not to be associated with mortality. As Seiler and authors excluded ESRD patients, the need for a study with ESRD-patients has been highlighted [23].

Moreover, recent experimental and clinical studies have challenged previous views on Klotho. Anour et al. failed to prove a physiological role for soluble and transmembrane Klotho in mineral metabolism and glucose homeostasis [24]. A study by Komaba et al. found no association between Klotho and the major players in arterial calcification in CKD-related mineral bone disorder PTH and FGF23 [25]. Furthermore, calcimimetic therapy in a hemodialysis patients was followed by very modest effects on Klotho levels, raising the question of whether these effects are biologically meaningful [25]. Levels of Klotho did not appear to correlate with residual renal function in a prevalent cohort of peritoneal dialysis patients [26], findings that were also seen in our cohort. No association between Klotho gene variants and the presence of valvular or vascular calcification was observed in the Framingham Offspring Cohort [27]. Taken together, the current data allow the conclusion that soluble Klotho levels do not reflect clinical outcome in patients with CKD.

FGF23 rather than Klotho more appropriately reflects cardiovascular risk. FGF23 is the earliest marker of deranged phosphate metabolism, increasing before PTH and phosphate levels and before vitamin D levels decrease [14]. Studies found FGF23 to be independently associated with mortality in hemodialysis patients [12], with ESRD development, cardiovascular events and mortality in CKD patients [13], [14]. In accordance with those previous studies, our study highlighted FGF23 as a valuable biomarker for assessment of cardiovascular risk in hemodialysis patients.

The FGF23 levels in this cohort appear lower compared to previous studies measuring C-terminal FGF23 in hemodialysis patients [12]. The cause is not completely clear. First, despite a good recovery in the dilution experiments using our samples, we cannot exclude some assay differences. Second, as FGF23 increases in the circulation with increasing phosphate load and vice versa [28], a further contributor could be differences in phosphate load. In our cohort, we may have had a high phosphate-clearance due to relatively long dialysis duration [29], a high percentages of high-flux dialysis membrane and hemodiafiltration (HDF) usage. All three modalities enhance phosphate clearance and possibly FGF23 removal itself. Previous studies showed that more intensive dialysis treatment can substantially lower FGF23 levels. Zaritzky et al achieved a median of 823 RU/ml in daily hemodialysis versus 2521 RU/ml in conventional hemodialysis patients [30]. Using HDF, Patrier et al achieved a 20% higher FGF23 clearance over high-flux hemodialysis only. Hemodialysis studies with FGF23 mostly do not give the exact information about dialysis treatment details hindering a direct comparison between studies. Importantly, although the FGF23 levels are lower in this study, its association with mortality is in accordance with previous work [12], [14].

Interestingly, the analytical concordance between Kainos and Millipore assays was in much closer agreement than between each of them and the Immutopics assay. A similar lack of agreement was found by Smith et al in a smaller group of hemodialysis patients [31]. The authors suggest that this might be due to differences in calibration. As the sample number where FGF23 levels were determined using different assays was not high in our study, further studies are warranted and careful interpretation of our results is recommended. Harmonisation of available assays would facilitate interpretation of studies where different assays are used.

Of note, patients with AF had lower Klotho levels than patients without AF. This effect remained significant even after the adjustment for cardiovascular comorbidities and approached significance in the fully adjusted model. Thus, high Klotho levels seem to exert a protective effect against AF. The absolute differences in Klotho levels between the patients groups with and without AF was small albeit significant. Small differences in sKlotho levels may be physiologically relevant. Relatively small increases in sKlotho levels were for example shown to induce overt changes in vascular tone of animal and human blood vessels in an experimental study by Six et al [32]. These dose dependent effects were shown to be due to sKlotho mediated intracellular reactive oxygen species (ROS) and nitric oxide (NO) production. We found no studies examining dose-dependent influence of ion channels by sKlotho. The physiological relevance of this relatively small difference in our study needs to be confirmed by further studies and must be regarded as a pilot hypothesis.

Concomitantly, patients with AF had higher FGF23 levels than patients without AF. The latter finding is in agreement with data from Seiler et al who found higher FGF23 levels in patients with AF in 885 subjects with normal and reduced kidney function undergoing elective coronary angiography [15].

A mechanistic explanation for the sinoatrial dysfunction might be arteriosclerotic changes caused by Klotho deficiency. But an even more plausible hypothesis is that Klotho might be essential for the function of ion channels which are responsible for the peacemaking activity in the sinoatrial node. In fact, a study by Takeshita et al. demonstrated specific expression of Klotho in the sinoatrial node peacemaker cells [19]. Furthermore, electrophysiological studies revealed significant sinus node dysfunction in Klotho knock-out mice [19]. Recent experimental research demonstrates that sKlotho can regulate the cell-surface abundance of ion channels such as Ca2+ and K+ channels [16]. The stimulation of the Ca2+ channel TRPV5, promoting the renal Ca2+ reabsorption by sKlotho was demonstrated in an animal study by Alexander et al [33]. Klotho has been shown to exhibit cardioprotective effects by downregulation of TRPC6 channels in the heart. But, these studies were performed only on isolated cardiac myocytes using supraphysiologic soluble Klotho concentrations. [17], [18], [34]. Low Klotho levels may therefore have led to sinoatrial dysfunction secondary to disturbed ion channels regulation in the cardiac peacemaker cells. Through protecting against AF, which is mostly attributed to senescence processes, Klotho appears to have anti-aging properties. With decreasing Klotho tertiles and therefore increasing AF prevalance, the treating physicians prescribed beta-, renin-angiotensin-aldosteron-blockers and statins more frequently (Table 1).

Klotho mainly derives from the renal tubular cells [4] both declining early in the CKD course [9], [35]. Correspondingly, Klotho levels in our healthy control group were higher than in CKD patients in the Study by Seiler et al [23] and the latter higher than in our hemodialysis patients (respective medians 690, 538 and 349 pg/ml). All levels measured using the same assay. But, Klotho levels were neither associated with CKD stages 2–4 [23] nor with residual kidney function in our patients. A possible explanation could be a reduced renal clearance in CKD. The fact that Klotho levels are maintained even with complete loss of kidney function suggests that sKlotho may be important for physiologic functions such as ion channels. The lack of association with mortality remains to be elucidated. One possibility is that Klotho level does not reflect membrane-bound Klotho function, the latter only being measurable by invasive methods.

Several limitations merit consideration. Firstly, the study was not designed to establish the exact cause of death. This limitation is common and may partly be due to the relatively low autopsy rate [14]. Due to this, we defined all-cause mortality rather than myocardial infarction (MI) as the primary end-point of the study with the knowledge that cardiovascular disease is the main contributor to mortality in ESRD [1]. Secondly, we did not define non-fatal MI as an end-point. Non-fatal MI rarely occurs in hemodialysis patients [36], the competing risk of cardiac death is observed to be several-fold greater than that of non-fatal MI [37] corresponding to the finding that coronary media calcifications rather than arterial plaques have a greater impact on mortality in dialysis patients [38], [39] and are the main autopsy finding [40]. We also did not record smoking and alcohol consumption habits at study enrolment. Thirdly, we cannot comment on unreported, asymptomatic or still undetected AF episodes. Furthermore, the association between low Klotho levels and AF was analysed in a cross-sectional manner and we have no data on AF incidence during the observational period. Nevertheless our hypothesis is supported, as the association between low Klotho levels and AF remained significant during analysis of the second independent blood sample, even after multiple adjustments. This hypothesis needs to be confirmed by further prospective studies. Experimental studies with systemic Klotho delivery in Klotho-deficient animals with AF could provide valuable information for understanding the mechanism underlying sinoatrial dysfunction and possibly lead to new therapeutic approaches in patients with AF. Another potential problem is that the three commercially available assays for the Klotho measurement differ in quality, as recently demonstrated by Heijboer et al [41]. However, we used the so far best validated assay with the lowest within- and between-run variation among the commercially available assays [41].

In conclusion, FGF23, but not Klotho levels, are associated with long-term mortality in ESRD-patients.

## Supporting Information

File S1 **Tables S1–S4 and Figure S1.** Table S1. Hazard Ratios (and 95% CIs) for Death per Standard Deviation of FGF23 and Klotho levels and according to the level tertiles. FGF23 and Klotho levels two weeks after enrolment. Table S2. Baseline characteristics and laboratory parameters according to presence or absence of atrial fibrillation. Table S3. Association of Klotho levels two weeks after enrolment with the presence of atrial fibrillation. Table S4. Regression analysis for Klotho tertiles two weeks after enrolment with the absence of atrial fibrillation. Figure S1. Dilution experiment for the Klotho and FGF23 assays. Eleven random samples were tested at sample: dilution ratios 1∶2 and 1∶4.
(DOC)
