# Supplementary material for: Prognostic Value and Link to Atrial Fibrillation of Soluble Klotho and FGF23 in Hemodialysis Patients
Source: PLoS One. 2014 Jul 3;9(7):e100688. doi: 10.1371/journal.pone.0100688 (PMC4084634; doi:10.1371/journal.pone.0100688)
Supplement: File S1 — Tables S1–S4 and Figure S1. Table S1. Hazard Ratios (and 95% CIs) for Death per Standard Deviation of FGF23 and Klotho levels and according to the level tertiles. FGF23 and Klotho levels two weeks after enrolment. Table S2. Baseline characteristics and laboratory parameters according to presence or absence of atrial fibrillation. Table S3. Association of Klotho levels two weeks after enrolment with the presence of atrial fibrillation. Table S4. Regression analysis for Klotho tertiles two weeks after enrolment with the absence of atrial fibrillation. Figure S1. Dilution experiment for the Klotho and FGF23 assays. Eleven random samples were tested at sample: dilution ratios 1∶2 and 1∶4. (DOC) [file pone.0100688.s001.doc]

Supporting Information

Table S1. Hazard Ratios (and 95% CIs) for Death per Standard Deviation of FGF23 and Klotho levels and according to the level tertiles. FGF23 and Klotho levels two weeks after enrolment.

| **Parameter** | **Crude** | | | **Model 1** ^*^ | | | **Model 2** ^†^ | | | **Model 3** ^‡^ | | |
| --- | --- | --- | --- | --- | --- | --- | --- | --- | --- | --- | --- | --- |
|  | HR | 95%CI | P value | HR | 95%CI | P value | HR | 95%CI | P value | HR | 95%CI | P value |
| Klotho (N=206) | 0.93 | 0.68-1.25 | 0.61 | 0.96 | 0.70-1.30 | 0.77 | 1.00 | 0.67-1.50 | 0.99 | 1.08 | 0.67-1.73 | 0.75 |
| Klotho ^§^ |  |  |  |  |  |  |  |  |  |  |  |  |
| Tertile 1 (N=68) | R |  |  | R |  |  | R |  |  | R |  |  |
| Tertile 2 (N=68) | 0.70 | 0.35-1.41 | 0.32 | 0.65 | 0.32-1.31 | 0.48 | 0.59 | 0.23-1.53 | 0.28 | 0.59 | 0.20-1.80 | 0.36 |
| Tertile 3 (N=70) | 0.71 | 0.35-1.43 | 0.34 | 0.78 | 0.38-1.57 | 0.48 | 0.61 | 0.22-1.53 | 0.36 | 0.68 | 0.22-2.12 | 0.51 |
| FGF23 (N=215) | 1.53 | 1.26-1.87 | <0.001 | 1.53 | 1.25-1.87 | <0.001 | 1.57 | 1.20-2.06 | 0.001 | 1.72 | 1.23-2.40 | 0.001 |
| FGF23 || |  |  |  |  |  |  |  |  |  |  |  |  |
| Tertile 1 (N=71) | R |  |  | R |  |  | R |  |  | R |  |  |
| Tertile 2 (N=71) | 1.13 | 0.82-3.27 | 0.16 | 1.14 | 0.54-2.40 | 0.73 | 1.41 | 0.54-3.70 | 0.16 | 1.49 | 0.52-5.59 | 0.46 |
| Tertile 3 (N=73) | 1.64 | 0.54-2.37 | 0.76 | 2.02 | 1.01-4.08 | 0.05 | 2.12 | 0.74-6.08 | 0.48 | 1.42 | 0.36-5.59 | 0.62 |

^*^ Model 1=demographics: adjusted for age, gender (male) and by dialysis center clustering.

^†^ Model 2=dialysis specific risk factors and comorbid conditions: adjusted for covariates in Model 1 plus dialysis vintage, systolic and diastolic blood pressure, body-mass index, vascular access on study enrolment (fistula, graft, catheter), coexisting conditions listed in Table 1 (coronary artery disease, valvular heart disease, atrial fibrillation, pulmonary hypertension, implantable cardioverter defibrillator carrier; diabetes mellitus, peripheral vascular disease, stroke, vasculitis, malignoma, chronic obstructive pulmonary disease), cause of renal failure (diabetic nephropathy, hypertensive nephropathy, glomerulonephritis, polycystic kidney disease, others / unknown), medication use listed in Table 1 (phosphate binders, vitamin D replacement, angiotensin converting enzyme inhibitors or angiotensin receptor blockers, beta blockers, aspirin, anticoagulant or clopidogrel, statin), pooled Kt/V.

^‡^ Model 3=fully adjusted model: adjusted for covariates in Model 2 plus parathyroid hormone, 25(OH)vitamin D, phosphate, calcium, albumin, hemoglobin, C-reactive protein, cholesterol.

^§^ Patients were categorized according to Klotho level tertiles two weeks after enrolment (1^st^ tertile <287 pg/ml, 2^nd^ tertile 287-407 pg/ml, 3^rd^ tertile >407 pg/ml).

^||^ Patients were categorized according to FGF23 level tertiles two weeks after enrolment (1^st^ tertile <131 RU/ml, 2^nd^ tertile 131-455 RU/ml, 3^rd^ tertile >455 RU/ml).

Abbreviations: FGF23, fibroblast growth factor 23; HR, hazard ratio; R, reference.

Table S2. Baseline characteristics and laboratory parameters according to presence or absence of atrial fibrillation.

|  | **Atrial fibrillation (N=54)** | **No atrial fibrillation (N=185)** | **P value** |
| --- | --- | --- | --- |
| Age (yr) | 76 ± 9 | 65 ± 14 | <0.001 |
| Male gender n (%) | 36 (67) | 117 (63) | 0.65 |
| Body-mass index ^*^ | 26 [23-29] | 26 [23-30] | 0.24 |
| Blood pressure (mmHg) |  |  |  |
| Systolic | 132 [122-139] | 136 [122-145] | 0.22 |
| Diastolic | 68 [62-74] | 69 [63-74] | 0.25 |
| Cause of renal failure n (%) |  |  |  |
| Diabetes mellitus | 13 (24) | 50 (27) | 0.65 |
| Hypertension | 6 (11) | 13 (7) | 0.33 |
| Glomerulonephritis | 17 (31) | 54 (29) | 0.75 |
| PKD | 2 (4) | 9 (5) | 0.72 |
| Others / unknown | 16 (30) | 59 (32) | 0.75 |
| Cardiac comorbidities n (%) |  |  |  |
| Coronary artery disease | 27 (50) | 46 (25) | <0.001 |
| Prior PCI/CABG | 17 (31) | 42 (23) | 0.18 |
| Valvular heart disease | 24 (44) | 37 (20) | <0.001 |
| Pulmonary hypertension | 6 (11) | 10 (5) | 0.14 |
| ICD implantation | 1 (2) | 3 (2) | 0.9 |
| Left atrium dilation (%) | 75 | 41 | <0.001 |
| Other comorbidities n (%) |  |  |  |
| Diabetes mellitus | 19 (35) | 71 (38) | 0.67 |
| PVD | 27 (50) | 53 (29) | 0.003 |
| Stroke | 11 (20) | 27 (15) | 0.31 |
| Vasculitis | 2 (4) | 6 (3) | 0.87 |
| Malignoma | 10 (19) | 24 (13) | 0.31 |
| COPD | 5 (9) | 14 (8) | 0.67 |
| Dialysis vintage (months) | 72 ± 60 | 55 ± 50 | 0.04 |
| Duration of dialysis session (hours) | 4.1 ± 0.3 | 4.2 ± 0.5 | 0.47 |
| Dialysis membrane n (%) |  |  |  |
| High-flux | 53 (98) | 166 (90) | 0.05 |
| Low-flux | 1 (2) | 19 (10) |  |
| Anuric patients n (%) | 31 (57) | 62 (34) | 0.002 |
| Residual diuresis (ml/24hours) | 0 [0-500] | 300 [0-1100] | 0.05 |
| Interdialytic weight gain (kg) | 1.9 [1.3-2.6] | 1.86 [1.25-2.44] | 0.43 |
| Shunt flow (ml/min) | 1193 ± 674 | 1172 ± 630 | 0.86 |
| Singe pool Kt / V | 1.59 [1.29-1.74] | 1.40 [1.40-1.73] | 0.55 |
| FGF23 (RU/ml) | 1569 ± 3108 | 681 ± 1378 | 0.003 |
| PTH (pg/ml) | 285 ± 205 | 242 ± 168 | 0.12 |
| 25(OH)vitamin D (ng/ml) | 27 [21-33] | 28 [21-35] | 0.45 |
| Phosphate (mmol/l) | 1.6 [1.3-1.8] | 1.6 [1.4-1.9] | 0.25 |
| Ca, serum (mmol/l) | 2.3 [2.2-2.4] | 2.3 [2.2-2.4] | 0.77 |
| Ca, dialysate (mmol/l) | 1.40 ± 0.14 | 1.45 ± 0.16 | 0.05 |
| K, serum (mmol/l) | 5.0 ± 0.7 | 5.0 ± 0.6 | 0.87 |
| K, dialysate (mmol/l) | 2.4 ± 0.6 | 2.4 ± 0.6 | 0.98 |
| AP (U/l) | 90 ± 34 | 91 ± 45 | 0.9 |
| Albumin (g/l) | 37 ± 5 | 37 ± 4 | 0.42 |
| Hemoglobin (g/dL) | 11.4 [11.2-11.8] | 11.5 [11.1-12.0] | 0.9 |
| C-reactive protein (mg/l) | 16 ± 16 | 11 ± 10 | 0.003 |
| Cholsterol (mg/dl) | 163 [134-185] | 167 [142-199] | 0.36 |
| Medication use n (%) |  |  |  |
| Phosphate binders | 47 (87) | 158 (85) | 0.9 |
| Vitamin D replacement | 54 (100) | 179 (97) | 0.27 |
| ACE-I or ARB | 27 (50) | 99 (54) | 0.57 |
| ß-Blockers | 42 (78) | 111 (60) | 0.02 |
| Aspirin | 22 (41) | 79 (44) | 0.73 |
| Anticoagulant or Clopidogrel | 37 (69) | 30 (16) | <0.001 |
| Statin | 25 (46) | 76 (41) | 0.48 |

Plus-minus values are means ± SD. Numbers with ranges in square brackets are medians and interquartile ranges. P values are for the comparisons between the three Klotho tertiles. To convert the values for calcium to milligrams per deciliter, multiply by 4.000. To convert the values for phosphate to milligrams per deciliter, multiply by 3.0969.

* The body-mass index is the weight in kilograms divided by the square of the height in meters.

**Abbreviations:** ACE-I, angiotensin-converting enzyme inhibitors; AF, atrial fibrillation; AP, alkaline phosphatase; ARB, angiotensin receptor blocker; Ca, calcium; COPD, chronic obstructive pulmonary disease; FGF23, fibroblast growth factor 23; ICD, implantable cardioverter defibrillator; IQR, interquartile range; K, potassium; PTFE, Polytetrafluorethylen; PTH, parathyroid hormone; PKD, polycystic kidney disease; PVD Peripheral vascular disease; T, Tertile; U, unit.

Table S3. Association of Klotho levels two weeks after enrolment with the presence of atrial fibrillation.

| **Parameter** | **Crude** | | | **Model 1** ^*^ | | | **Model 2** ^†^ | | | **Model 3** ^‡^ | | |
| --- | --- | --- | --- | --- | --- | --- | --- | --- | --- | --- | --- | --- |
|  | OR | 95%CI | P value | OR | 95%CI | P value | OR | 95%CI | P value | OR | 95%CI | P value |
| Klotho ^§^ (pg/mL) | 0.59 | 0.52-0.68 | <0.001 | 0.39 | 0.27-0.58 | <0.001 | 0.39 | 0.25-0.60 | <0.001 | 0.53 | 0.32-0.88 | 0.01 |
| Age (years) |  |  |  | 1.02 | 1.03-1.12 | 0.04 | 1.00 | 0.99-1.02 | 0.75 | 1.05 | 1.01-1.10 | 0.02 |
| Gender (male) |  |  |  | 1.05 | 0.51-2.12 | 0.91 | 0.89 | 0.39-2.03 | 0.78 | 1.48 | 0.51-4.31 | 0.47 |
| DM |  |  |  |  |  |  | 0.68 | 0.27-1.75 | 0.43 | 0.67 | 0.25-1.78 | 0.42 |
| CVD |  |  |  |  |  |  | 2.58 | 1.15-5.78 | 0.02 | 2.69 | 1.09-6.60 | 0.03 |
| VHD |  |  |  |  |  |  | 1.85 | 0.80-4.27 | 0.15 | 1.54 | 0.60-3.95 | 0.37 |
| PAD |  |  |  |  |  |  | 1.86 | 0.75-4.59 | 0.18 | 1.81 | 0.68-4.80 | 0.24 |
| Stroke |  |  |  |  |  |  | 1.70 | 0.64-4.46 | 0.29 | 1.91 | 0.66-5.52 | 0.24 |
| Anuria |  |  |  |  |  |  | 2.03 | 0.93-4.45 | 0.08 | 3.38 | 1.32-8.69 | 0.01 |
| PTH (pg/ml) |  |  |  |  |  |  |  |  |  | 1.00 | 1.00-1.00 | 0.40 |
| FGF23 ^§^ (RU/ml) |  |  |  |  |  |  |  |  |  | 2.04 | 0.63-6.61 | 0.24 |
| Phosphate (mmol/l) |  |  |  |  |  |  |  |  |  | 0.35 | 0.09-1.35 | 0.13 |
| Ca, serum (mmol/l) |  |  |  |  |  |  |  |  |  | 0.79 | 0.04-14.4 | 0.88 |
| Ca, dialysate (mmol/l) |  |  |  |  |  |  |  |  |  | 0.03 | 0.002-0.52 | 0.02 |
| K, serum (mmol/l) |  |  |  |  |  |  |  |  |  | 0.80 | 0.41-1.56 | 0.52 |
| K, dialysate (mmol/l) |  |  |  |  |  |  |  |  |  | 1.52 | 0.75-3.09 | 0.25 |
| Albumin (g/l) |  |  |  |  |  |  |  |  |  | 1.04 | 0.91-1.19 | 0.58 |
| Hb (g/dl) |  |  |  |  |  |  |  |  |  | 1.04 | 0.57-1.90 | 0.89 |
| CRP (mg/l) |  |  |  |  |  |  |  |  |  | 1.00 | 0.97-1.04 | 0.90 |
| Cholesterol (mg/dl) |  |  |  |  |  |  |  |  |  | 1.00 | 0.99-1.01 | 0.75 |
| TSH (mU/l) |  |  |  |  |  |  |  |  |  |  |  |  |

^*^ Model 1: adjusted for age and gender (male).

^†^ Model 2: adjusted for covariates in Model 1 plus cardiovascular comorbidities and anuria.

^‡^ Model 3: adjusted for covariates in Model 2 plus laboratory results for mineral metabolism, calcium dialysate, potassium serum and dialysate, inflammation, cholesterol, hemoglobin and thyroid stimulating hormone.

^§^ per standard deviation

Abbreviations: CI, confidence interval; CRP, C-reactive protein; CVD, cardiovascular disease; DM, diabetes mellitus; FGF23, fibroblast growth factor 23; Hb, hemoglobin; PAD, peripheral artery disease; OR, odds ratio; VHD, valvular heart disease.

Table S4. Regression analysis for Klotho tertiles two weeks after enrolment with the absence of atrial fibrillation.

|  | **Klotho Tertile 1, N=68 (<287 pg/ml)** | **Klotho Tertile 2, N=68(287- 407 pg/ml)** | | **Klotho Tertile 3, N=70 (>407 pg/ml)** | |
| --- | --- | --- | --- | --- | --- |
|  |  | OR (95%CI) | P value | OR (95%CI) | P value |
| Crude | Reference | 3.25 (1.86-5.69) | <0.001 | 9.00 (4.12-19.65) | <0.001 |
| Model 1 ^*^ | Reference | 2.03 (0.98-4.12) | 0.06 | 5.75 (2.35-14.05) | <0.001 |
| Model 2 ^†^ | Reference | 2.21 (0.98-4.96) | 0.06 | 5. 53 (2.10-14.55) | 0.001 |
| Model 3 ^‡^ | Reference | 2.22 (0.80-6.19) | 0.13 | 5.12 (1.53-17.12) | 0.008 |

^*^ Model 1: adjusted for age and gender.

^†^ Model 2: adjusted for covariates in Model 1 plus diabetes mellitus, coronary artery disease, valvular heart disease, peripheral vascular disease, stroke and anuria.

^‡^ Model 3: adjusted for covariates in Model 2 plus parathyroid hormone, fibroblast growth factor 23, calcium, phosphate, albumin, calcium dialysate, potassium serum and dialysate,hemoglobin, C-reactive protein cholesterol, and thyroid stimulating hormone.

Abbreviations: CI, confidence interval; OR, odds ratio.

**Figure S1. Dilution experiment for the Klotho and FGF23 assays.**

Eleven random samples were tested at sample:dilution ratios 1:2 and 1:4.


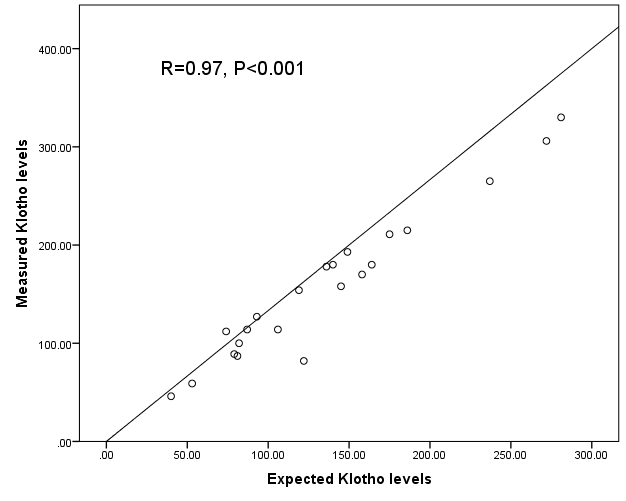


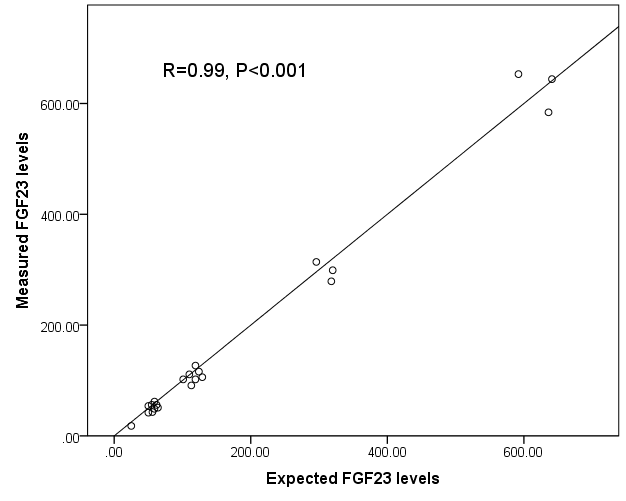


Abbreviations: FGF23, fibroblast growth factor 23
